# An Ameliorated Prediction of Drug–Target Interactions Based on Multi-Scale Discrete Wavelet Transform and Network Features

**DOI:** 10.3390/ijms18081781

**Published:** 2017-08-16

**Authors:** Cong Shen, Yijie Ding, Jijun Tang, Xinying Xu, Fei Guo

**Affiliations:** 1 School of Computer Science and Technology, Tianjin University, Tianjin 300350, China; congshen@tju.edu.cn (C.S.); wuxi_dyj@tju.edu.cn (Y.D.); 2 Tianjin University Institute of Computational Biology, Tianjin University, Tianjin 300350, China; 3 Department of Computer Science and Engineering, University of South Carolina, Columbia, SC 29208, USA; 4 College of Information Engineering, Taiyuan University of Technology, Taiyuan 030024, Shanxi, China; xuxinying@tyut.edu.cn

**Keywords:** drug–target interactions, discrete wavelet transform, network property, support vector machine

## Abstract

The prediction of drug–target interactions (DTIs) via computational technology plays a crucial role in reducing the experimental cost. A variety of state-of-the-art methods have been proposed to improve the accuracy of DTI predictions. In this paper, we propose a kind of drug–target interactions predictor adopting multi-scale discrete wavelet transform and network features (named as DAWN) in order to solve the DTIs prediction problem. We encode the drug molecule by a substructure fingerprint with a dictionary of substructure patterns. Simultaneously, we apply the discrete wavelet transform (DWT) to extract features from target sequences. Then, we concatenate and normalize the target, drug, and network features to construct feature vectors. The prediction model is obtained by feeding these feature vectors into the support vector machine (SVM) classifier. Extensive experimental results show that the prediction ability of DAWN has a compatibility among other DTI prediction schemes. The prediction areas under the precision–recall curves (AUPRs) of four datasets are 0.895 (Enzyme), 0.921 (Ion Channel), 0.786 (guanosine-binding protein coupled receptor, GPCR), and 0.603 (Nuclear Receptor), respectively.

## 1. Introduction

Although the PubChem database [1] has stored millions of chemical compounds, the number of compounds having target protein information are limited. Drug discovery (finding new drug–target interactions, DTIs) requires much more cost and time via biochemical experiments. Hence, some efficient computational methods for predicting potential DTIs are used to cover the shortage of traditional experimental methods. There are three categories of the DTIs prediction approaches: molecular docking, matrix-based, and feature vector-based methods. Cheng et al. [2] and Rarey et al. [3] developed molecular docking methods, which were based on the crystal structure of the target binding site (3D structures). Docking simulations quantitatively estimate the maximal affinity achievable by a drug-like molecule, and these calculated values correlate with drug discovery outcomes. However, docking simulations depend on the spatial structure of targets and are usually time-consuming because of the screening technique. In contrast to docking methods, the other two kinds of computational methods (matrix-based and feature vector-based methods) can achieve the large-scale prediction of DTIs.

Compared with molecular docking, matrix-based methods of chemical structure similarity are more popular. Many matrix-based approaches are becoming popular in the area of DTI predicition. The bipartite graph learning (BGL) [4] model was firstly proposed by Yamanishi et al. They developed a new supervised method to infer unknown DTIs by integrating chemical space and genomic space into a unified space. Bleakley and Yamanishi et al. [5] raised the bipartite local model (BLM) to solve the DTI prediction problem in chemical and genomic spaces, and applied the bipartite model to transform prediction into a binary classification [5]. Mei et al. [6] improved the BLM with neighbor-based interaction-profile inferring (BLM-NII). The NII strategy inferred label information or training data from neighbors when there was no training data readily available from the query compound/protein itself. Laarhoven et al. designed kernel regularized least squares (RLS), in which they defined Gaussian interaction profile (GIP) kernels on the profiles of drugs and targets to predict DTIs [7]. Xia et al. raised Laplacian regularized least square based on interaction network (NetLapRLS) [8] to improve the prediction performance of RLS. Zheng et al. built a DTI predictor with collaborative matrix factorization (CMF) [9], which can incorporate multiple types of similarities from drugs and those from targets at once. Laarhoven et al. [10] also proposed weighted nearest neighbor with Gaussian interaction profile kernels (WNN-GIP) to predict DTIs. The WNN constructed an interaction score profile for a new drug compound using chemical and interaction information about known compounds in the dataset. Another matrix factorization-based method—kernelized Bayesian matrix factorization with twin kernels (KBMF2K) [11]—was proposed by Gönen, M. The novelty of KBMF2K came from the joint Bayesian formulation of projecting drug compounds and target proteins into a unified subspace using the similarities and estimating the interaction network in that subspace. Neighborhood regularized logistic matrix factorization (NRLMF) was raised by Liu et al. [12]. NRLMF focused on modeling the probability that a drug would interact with a target by logistic matrix factorization, where the properties of drugs and targets were represented by drug-specific and target-specific latent vectors, respectively. Nevertheless, the drawback of pairwise kernel method is the high computational complexity on the occasion of a large numbers of samples. In addition, matrix-based methods did not consider the physical and chemical properties of the target protein. These properties reflect some particular relationship between targets and the molecular structure of drugs.

To handle the above problem, other machine learning approaches of feature vector-based method was raised. Cao et al. firstly proposed several works to predict DTIs via drug (molecular fingerprint), target (sequence descriptors), and network information [13,14]. They used composition (C), transition (T), and distribution (D) and Molecular ACCess System (MACCS) fingerprint to describe target sequence and drug molecule, respectively. The above features were fed into random forest (RF) to detect DTIs.

In this article, we propose a new DTI predictor based on signal compression technology. The target sequence can be regarded as biomolecule signal of a cell. To further extract effective features from the target sequence, we utilize discrete wavelet transform (DWT) as a spectral analysis tool to compress the signal of the target sequence. According to Heisenberg’s uncertainty principle, the velocity and location of moving quanta cannot be determined at the same time. Similarly, in a time–frequency coordinate system, the frequency and location of a signal cannot be determined at the same time. Wavelet transform can be based on the scale of the transformation and offset in different frequency bands, given different resolution. This is an effective scenario in practice. We also use MACCS fingerprint to describe the drug. Further more, network feature provides the relationship between drug–target pairs. Many models (e.g., BLM, BLM-NII, NetLapRLS, CMF, KBMF2K, NRLMF, and Cao’s work [14]) were built with network information. Therefore, our feature contains sequence (DWT feature), drug (MACCS feature), and network (net feature). Moreover, we combine the above three types of features with support vector machine (SVM) and feature selection (FS) to develop a predictor of DTIs. We evaluate our method on four benchmark datasets including Enzyme, Ion Channel, guanosine-binding protein coupled receptor (GPCR), and Nuclear receptor. The result shows that our method achieves better prediction performance than outstanding approaches.

## 2. Results

We evaluated our method (DAWN) on balanced DTI datasets, described by Cao’s work [14]. We analyzed the performance of features (including MACCS, DWT, and net feature). Then, we compared DAWN with other outstanding methods, including BLM [5], RLS [7], BGL [4], NetLapRLS [8], and Cao’s work [14]. In addition, we also tested DAWN on imbalanced DTI datasets, compared with NetLapRLS [8], BLM-NII [6], CMF [9], WNN-GIP [10], KBMF2K [11], and NRLMF [12]. We found that DAWN achieved better values of AUCs.

### 2.1. Dataset

To evaluate the performance and scalability of our method, we adopted enzyme, ion channels, GPCR, and nuclear receptors used by Yamanishi et al. [4] as the gold standard datasets. These datasets come from the Kyoto Encyclopedia of Genes and Genomes (KEGG) database [15]. The information of drug–target interactions comes from KEGG BRITE [15], BRENDA [16], Super Target [17], and DrugBank databases [18]. Table 1 presents some quantitative descriptors about the golden datasets, including the number of drugs (*n*), number of targets (*m*), number of interactions, and ratio of *n* to *m*.

#### 2.1.1. Balanced Dataset

In Cao’s study [14], all real drug–target interaction pairs were used as the positive samples. For negative examples, they selected random, unknown interacting pairs from these drug and protein molecules. DAWN was tested on Cao’s four balanced benchmark datasets (including Enzyme, Ion channels, GPCRs, and Nuclear receptors).

#### 2.1.2. Imbalanced Dataset

The gold standard datasets only contain positive examples (interaction pairs). Hence, non-interaction drug–target pairs are considered as negative examples. Because the number of non-interaction pairs is larger than interaction pairs, the ratio between majority and minority examples is much greater than 1.

### 2.2. Evaluation Measurements

Three parameters were adopted as criteria: overall prediction accuracy (ACC), sensitivity (SN), and specificity (Spec).

- Accuracy:
  (1) $$\;ACC=\frac{TP+TN}{TP+FP+TN+FN}$$
- Sensitivity or Recall:
  (2) $$\;SN=\frac{TP}{TP+FN}$$
- Specificity:
  (3) $$\;Spec=\frac{TN}{TN+FP}$$

TP represents the number of positive samples predicted correctly. Similarly, we have TN, FP and FN, which represent the number of negative samples predicted correctly, the number of negative samples predicted as positive, and the positive samples predicted as negative, respectively.

In signal detection theory, a receiver operating characteristic (ROC), or simply ROC curve, is a graphical plot illustrating the performance of a binary classifier system as its varied discrimination threshold. A ROC curve can be used to illustrate the relation between sensitivity and specificity.

Area under the precision–recall curve (PRC) (AUPR) is an average of the precision weighted by a given threshold probability. We employed both ROC and the area under the precision–recall curve (PRC), because the representation of PRC is more effective than ROC on highly imbalanced or skewed datasets. Area under the ROC curve (AUC) and AUPR can quantitatively describe sensitivity against specificity and precision against recall, respectively.

### 2.3. Experimental Results on Balanced Datasets

#### 2.3.1. Performance Analysis of Feature

In order to analyze the performance of MACCS, DWT, and net features, we tested these features on four balanced datasets (each set contains 10 balanced subsets) through five-fold cross-validation. Results of DWT + MACCS, DWT + MACCS (with FS), DWT + NET + MACCS, and DWT + NET + MACCS (with FS) are shown in Table 2. Because the datasets are balanced, the evaluation of ACC or AUC can measure overall performance. DWT + NET + MACCS (with FS) had the best performance of ACC on Enzyme (0.938), IC (0.943), GPCR (0.890), and Nuclear receptor (0.860), respectively. The performance (AUC) of DWT + NET + MACCS (Enzyme: 0.977, IC: 0.978, GPCR: 0.934, Nuclear receptor: 0.866) was better than DWT + MACCS (Enzyme: 0.925, IC: 0.929, GPCR: 0.872, Nuclear receptor: 0.816). The feature DWT + NET + MACCS indeed improved the prediction performance by adding network information. In addition, the performance (AUC) of DWT + NET + MACCS (with FS) (Enzyme: 0.980, IC: 0.983, GPCR: 0.950, Nuclear receptor: 0.931) was better than DWT + NET + MACCS (without FS) (Enzyme: 0.977, IC: 0.978, GPCR: 0.934, Nuclear receptor: 0.866).

**Figure 1:**
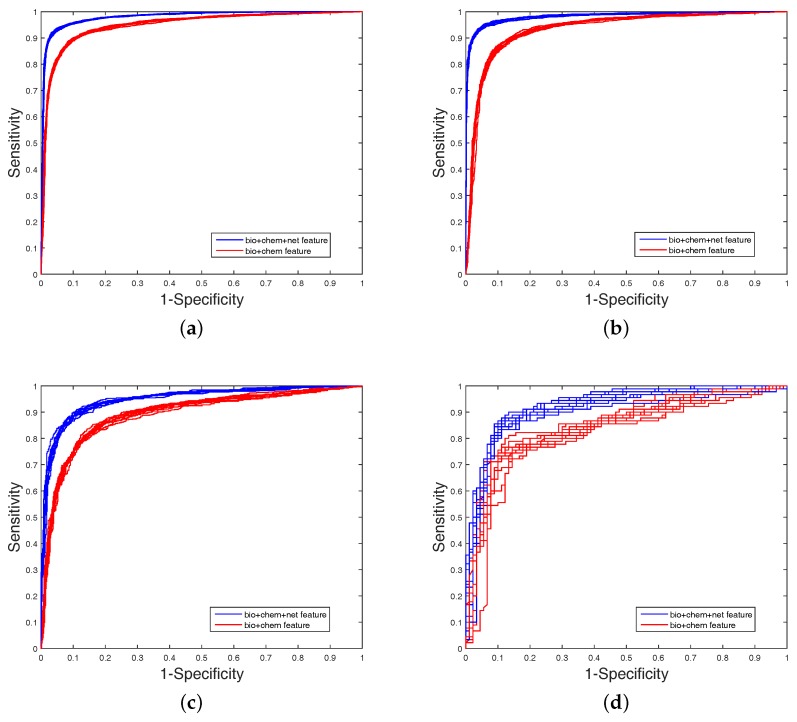
The area under the Receiver Operating characteristic Curve (ROC) values obtained on balanced datasets (with FS). The blue curve is the combined feature of MACCS (chem), DWT (bio), and net. The red curve is the combined feature of MACCS (chem) and DWT (bio); (**a**) Enzyme’s ROC curve with network feature; (**b**) IC ’s ROC curve with network feature; (**c**) GPCR’s ROC curve with network feature; (**d**) Nuclear receptor’s ROC curve with network feature.

**Figure 2:**
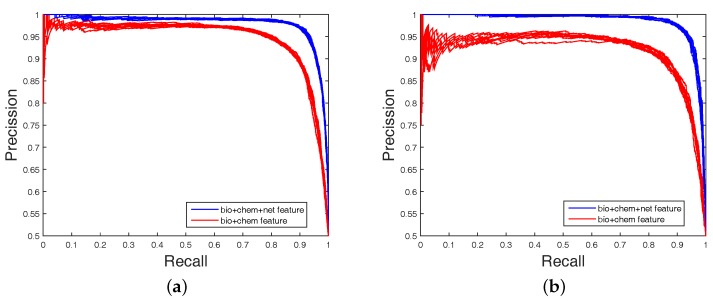

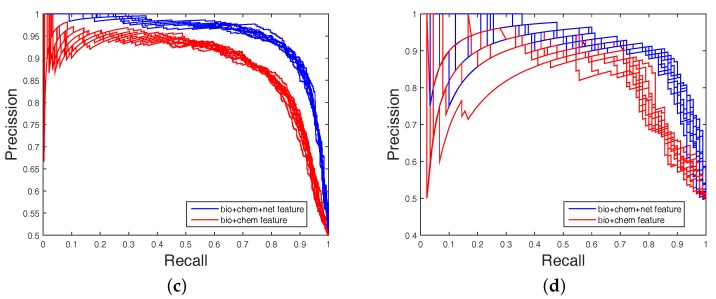
The area under the precision–recall (PR) curve (AUPR) values obtained on balanced datasets (with FS). The blue curve is the combined feature of MACCS (chem), DWT (bio), and net. The red curve is the combined feature of MACCS (chem) and DWT (bio); (**a**) Enzyme’s PR curve with network feature; (**b**) IC’s PR curve with network feature; (**c**) GPCR’s PR curve with network feature; (**d**) Nuclear receptor’s PR curve with network feature.

It is clear that FS plays a key role in elevating the prediction of our method. The FS can enhance generalization by reducing the overfitting. Obviously, the performance of DWT + NET + MACCS (with FS) can be seen from Figure 1 and Figure 2. Network topology can be a useful supplement to improve prediction effect.

#### 2.3.2. Comparing with Existing Methods

On the balanced datasets [14], we compare DAWN with other common methods by five-fold cross validation. These methods contain BLM [5], RLS [7], BGL [4], NetLapRLS [8] and Cao’s work [14]. The detailed results are listed in Table 3. DAWN achieved the best values of AUCs on Enzyme (0.980) and Nuclear receptor (0.931), respectively. Although the AUC value of DAWN on Ion channel and GPCR datasets were not higher than Cao’s work [14] and BLM, we still have a competitive prediction rate. Recapitulating about the aforementioned description, DAWN has a competitive ability among these works.

### 2.4. Experimental Results on Imbalanced Datasets

**Figure 3:**
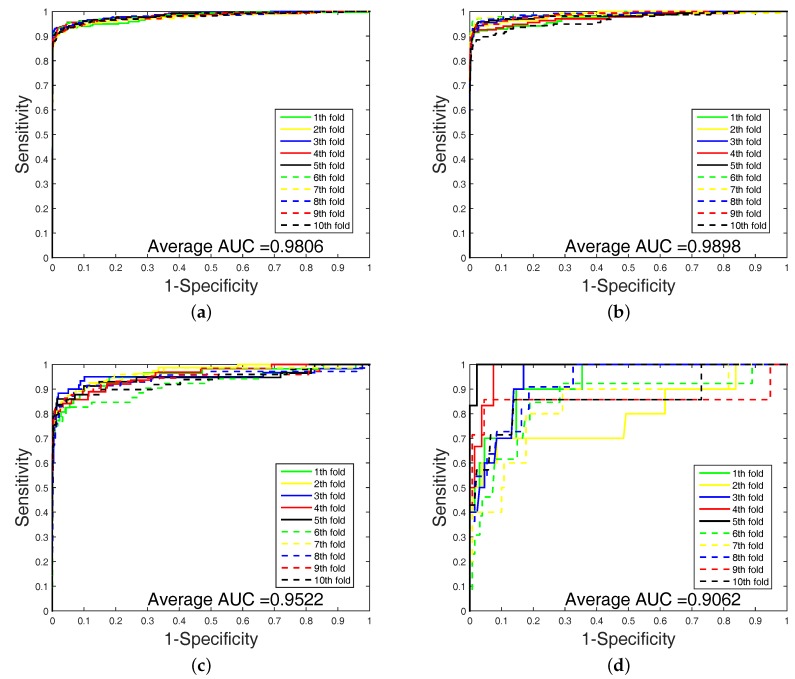
ROC of imbalanced datasets by 10-fold cross-validation; (**a**) Enzyme’s ROC curve with network feature; (**b**) IC’s ROC curve with network feature; (**c**) GPCR’s ROC curve with network feature; (**d**) Nuclear receptor’s ROC curve with network feature.

**Figure 4:**
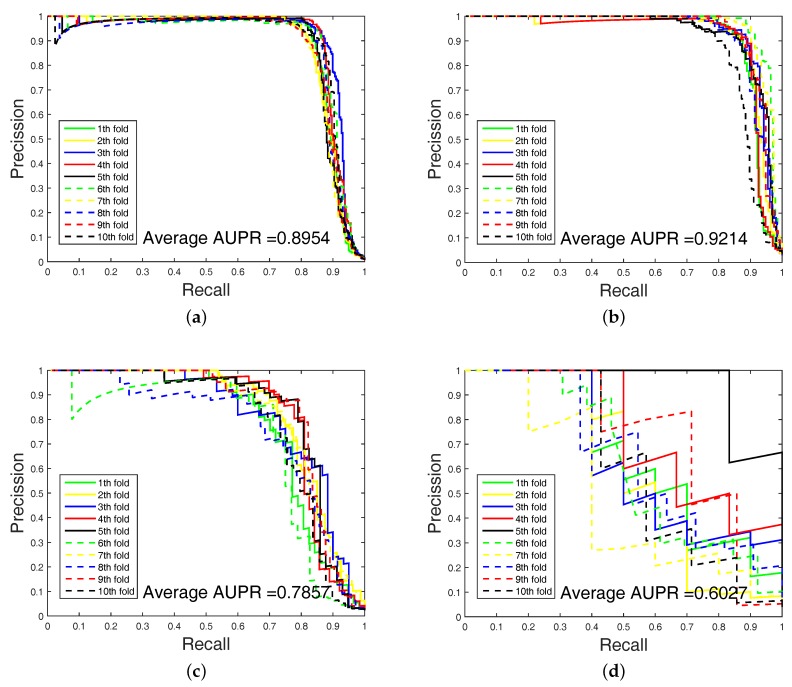
AUPR of imbalanced datasets by 10-fold cross-validation. (**a**) Enzyme’s PR curve with network feature. (**b**) IC’s PR curve with network feature. (**c**) GPCR’s PR curve with network feature. (**d**) Nuclear receptor’s PR curve with network feature.

In order to highlight the advantage of our method, we also tested DAWN on the imbalanced datasets of DTIs by 10-fold cross validation. DAWN was compared with NetLapRLS [8], BLM-NII [6], CMF [9], WNN-GIP [10], KBMF2K [11], and NRLMF [12]. The detailed results are listed in Table 4. Because the datasets are imbalanced, the evaluation of AUC and AUPR were both used to measure overall performance. DAWN achieved average AUCs of 0.981, 0.990, 0.952, and 0.906, and the AUPR values of DAWN were 0.895, 0.921, 0.786, and 0.603 on Enzyme, Ion channel, GPCR, and Nuclear receptor, respectively. The AUC value of DAWN on the Enzyme dataset was 0.981 and AUPR was 0.895, and only the NRLMF (AUC: 0.987, AUPR: 0.892) method was comparable. On Ion channel and GPCR datasets, we also had best or second-best results. For AUPR value on Nuclear receptor, NRLMF was higher than DAWN. The Nuclear receptor dataset is smaller than the other three datasets. The size of the dataset might be a reason for DAWN’s performance. Therefore, the DAWN method that adopted the mean of DWT was not as effective as larger datasets. However, among methods in Table 4, none could give markedly higher prediction performance on all four datasets in both AUC and AUPR. Therefore, it is fair to claim that our strategy has comparable performance. Further, Figure 3 and Figure 4 show the curves of AUC and AUPR on imbalanced datasets through 10-fold cross validation. Related datasets, codes, and figures of our algorithm are available at https://github.com/6gbluewind/DTIDWT.

### 2.5. Predicting New DTIs

In this experiment, the balanced DTIs were set as training data sets. We ranked the remaining non-interacting pairs and selected the top five non-interacting pairs as predicted interactions. We utilized four well-known biological databases (including ChEMBL (C) [19], DrugBank (D) [18], KEGG (K) [15] and Matador (M) [20]) as references to verify whether or not the predicted new DTIs are true. The predicted novel interactions by DAWN can be ranked based on the interaction probabilities, which are shown in Table 5. The potential DTIs may be present in one or several databases. For example, the secondly ranked DTI of GPCR (D00563: hsa3269) belongs to DrugBank and Matador databases. In addition, the DTI databases (the above four databases) are still being updated, and the accuracy of identifying new DTIs by DAWN may be increased.

## 3. Discussion

In this paper, we proposed a new DTIs predictor based on signal compression technology. We encoded the drug molecule by a substructure fingerprint with a dictionary of substructure patterns. Moreover, we applied the DWT to extract features from target sequences. At last, we concatenated the target, drug, and network features to construct predictive model of DTIs.

To evaluate the performance of our method, the DTIs model was compared to other state-of-the-art DTIs prediction methods on four benchmark datasets. DAWN achieved average AUCs of 0.981, 0.990, 0.952, and 0.906, and the AUPR values of DAWN were 0.895, 0.921, 0.786, and 0.603 on Enzyme, Ion channel, GPCR, and Nuclear receptor, respectively. Although our result using feature selection could be a kind of ameliorated prediction, the imbalanced problem of DTIs prediction is not solved very well. SVM is poor on imbalanced data. The AUPR value of DAWN is low on the Nuclear receptor dataset.

## 4. Materials and Methods

To predict DTIs by machine learning methods, one challenge is to extract effective features from the target protein, drug, and the relationship between drug–target pairs. Considering that DTIs depend on the molecular properties of the drug and the physicochemical properties of target, we use MACCS fingerprints (Open Babel 2.4.0 Released, OpenEye Scientific Software, Inc., Santa Fe, New Mexico, United States) to represent the drug, and extract biological features from the target via DWT. In addition, the net feature describes the topology information of the DTIs network. We utilize the above features to train the SVM predictor (LIBSVM Version 3.22, National Taiwan University, Taiwan, China) for detecting DTIs.

### 4.1. Molecular Substructure Fingerprint of Drug

To encode the chemical structure of the drug, we utilize MACCS fingerprints with 166 common chemical substructures. These substructures are defined in the Molecular Design Limited (MDL) system, which can be found from OpenBabel (http://openbabel.org). The MACCS feature is encoded by a binary bits vector, which shows the presence (1) or absence (0) of some specific substructures in a molecule. Please refer to the relevant literature [13,14] for details.

### 4.2. Biological Feature of Target

#### 4.2.1. Six Physicochemical Properties of Amino Acids

The target sequence can be denoted by $seq=\{r_{1},r_{2},\cdots ,r_{i},\cdots ,r_{L}\}$, where 1≤i≤L. $r_{i}$ is the *i*-th residue of sequence seq, and *L* is the length of sequence seq. In addition, for ease of calculation about feature representation, we select six kinds of physicochemical properties for 20 amino acid types as original target features [21,22,23,24]. More specifically, they are hydrophobicity (H), volumes of side chains of amino acids (VSC), polarity (P1), polarizability (P2), solvent-accessible surface area (SASA) and net charge index of side chains (NCISC), respectively. Values of all kinds of amino acid are shown in Table 6.

For the sake of facilitating the dealing with the datasets, the amino acid residues are translated and normalized according to Equation (4).

(4) $$P_{ij}^{\prime }=\frac{P_{ij}-P_{j}}{S_{j}}\left(j=1,2,\ldots ,6;\;i=1,2,\ldots ,20\right)$$

where $P_{i,j}$ and $P_{j}$ indicate the value of the *j*-th descriptor of amino acid type *i* and the mean of 20 amino acid types of descriptor value *j*, respectively, standard deviation (SD) corresponding to $S_{j}$.

Each target sequence can be translated into six vectors with each amino acid represented by normalized values of six descriptors. Thus, the seq can be represented as physicochemical matrix $X=\left[x_{1},...,x_{ch},...,x_{6}\right],X\in R^{L\times 6},x_{ch}\in R^{L\times 1},ch=1,2,...,6$.

#### 4.2.2. Discrete Wavelet Transform

Discrete wavelet transform (DWT) with its inversion formula was established by physical intuition and practical experience of signal processing [25].

If a signal or a function can be represented as Equation (5), then the signal or function has a linear decomposition. If the formula of expansion is unique, then the set of expansion can be said as a group of basis. If this group of basis is orthogonal or represented as Equation (6), then the coefficient can be computed by inner product as Equation (7).

(5) $$f\left(t\right)=\sum_{\ell }a_{\ell }\psi_{\ell }\left(t\right),$$

(6) $$\left(\psi_{k}\left(t\right),\psi_{\ell }\left(t\right)\right)=\int \psi_{k}\left(t\right)\psi_{\ell }\left(t\right)dt=0,k\neq \ell ,$$

(7) $$a_{k}=\left(f\left(t\right),\psi_{k}\left(t\right)\right)=\int f\left(t\right)\psi_{k}\left(t\right)dt,$$

where *ℓ* and *k* are the finite or infinite integer indexes, $a_{\ell }$ and $a_{k}$ are the real coefficients of the expansion, and $\psi_{\ell }\left(t\right)$ and $\psi_{k}\left(t\right)$ are the set of real functions.

For wavelet expansion, we can construct a system with two parameters, then the formula can be transferred as Equation (8):

(8) $$f\left(t\right)=\sum_{k}\sum_{j}a_{j,k}\psi_{j,k}\left(t\right),$$

where *j* and *k* are integer index, and $\psi_{j,k}\left(t\right)$ is wavelet function, which generally forms a group of orthogonal basis.

The expansion coefficient set $a_{j,k}$ is known as the discrete wavelet transform (DWT) of f(t). Nanni et al. proposed an efficient algorithm to perform DWT by assuming that the discrete signal f(t) is $x_{ch}\left(n\right)$.

(9a) $$\begin{array}{cc} y_{l,high,ch}\left(n\right) & =\sum_{k=1}^{L}\left[x_{ch}\left(k\right)\cdot h\left(2n-k\right)\right] \end{array}$$

(9b) $$\begin{array}{cc} y_{l,low,ch}\left(n\right) & =\sum_{k=1}^{L}\left[x_{ch}\left(k\right)\cdot g\left(2n-k\right)\right] \end{array}$$

where *h* and *g* refer to high-pass filter and low-pass filter, *L* is the length of discrete signal, $y_{l,low,ch}\left(n\right)$ is the approximate coefficient (low-frequency components) of the signal, l(l=1,2,3,4) is the decomposition level of DWT, ch(ch=1,2,3,4,5,6) is the physicochemical index, and $y_{l,low,ch}\left(n\right)$ is the detailed coefficient (high-frequency components).

**Figure 5:**
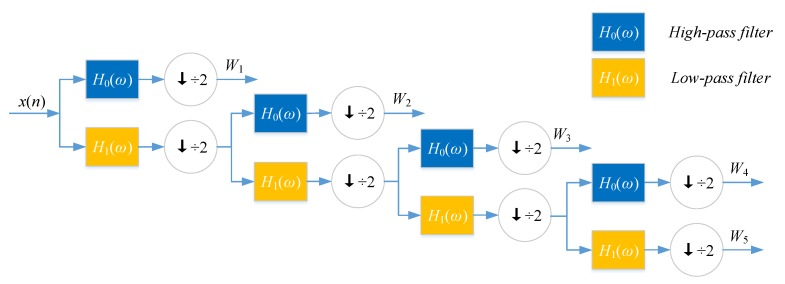
Wavelet decomposition tree.

DWT can decompose discrete sequences into high- and low-frequency coefficients. Nanni et al. [26] substituted each amino acid of the protein sequence with a physicochemical property. Then, the protein sequence was encoded as a numerical sequence. DWT compresses discrete sequence and removes noise from the origin sequence. Different decomposition scales with discrete wavelet have different results for representing the sequence of the target protein. They used 4-level DWT and calculated the maximum, minimum, mean, and standard deviation values of different scales (four levels of both low- and high-frequency coefficients). In addition, high-frequency components are more noisy while low-frequency components are more critical. Therefore, they extracted the beginning of the first five Discrete Cosine Transform (DCT) coefficients from the approximation coefficients. We utilize Nanni’s method to describe the sequence of the target protein. The schematic diagram of a 4-level DWT is shown in Figure 5.

### 4.3. Drug–Target Associations from Network

State-of-the-art works such as BLM [5], BLM-NII [6], NetLapRLS [8], CMF [9], KBMF2K [11], NRLMF [12], and Cao’s work [14] used DTI network topology information to improve the prediction performance. Therefore, we also consider utilizing net feature to build a DTI predictor.

The DTI network can be conveniently regarded as a bipartite graph. In the network, each drug is associated with $n_{t}$ targets, and each target is associated with $n_{d}$ drugs. Excluding target $T_{j}$ itself, we make a binary vector of all other known targets of $D_{i}$ in the bipartite network, as well as a separate list of targets not known to be targeted by $D_{i}$. Known and unknown targets are labeled by 1 and 0, respectively. For drug $D_{i}$, we get $\left(n_{t}-1\right)$-dimensional binary vector. Similarly, we also get $\left(n_{d}-1\right)$-dimensional binary vector of target $T_{j}$. Thus, we can get a $\left[\left(n_{d}-1\right)+\left(n_{t}-1\right)\right]$-dimensional vector for describing net feature.

### 4.4. Feature Selection and Training SVM Model

Not all features are useful for DTIs prediction. Therefore, we apply support vector machine recursive feature elimination and correlation bias reduction (SVM-RFE+CBR) [27,28] to select the important features of DTIs. The SVM-RFE+CBR can estimate the score of importance for each dimensional feature. We rank these features (including MACCS feature, DWT feature, and net feature) by the scores in descending order. Then, we select an optimal feature subset in top *k* ranked manner to predict DTIs.

Support vector machine (SVM) was originally developed by Vapnik [29] and coworkers, and has shown a promising capability to solve a number of chemical or biological classification problems. SVM and other machine learning algorithms (e.g., random forest, RF, k-nearest neighbor, kNN, etc.) are widely used in computational biology [30,31,32,33]. SVM performs classification tasks by constructing a hyperplane in a multidimensional space to differentiate two classes with a maximum margin. The input data of SVM is defined as {$x_{i},y_{i}$}, i=1,2,...,N, feature vector $x_{i}\in R^{n}$ and labels $y_{i}\in \left\{+1,-1\right\}$.

The classification decision function implemented by SVM is shown as Equation (10).

(10) $$f\left(x\right)=sgn\{\sum_{i=1}^{N}y_{i}\alpha_{i}\cdot K\left(x,x_{i}\right)+b\}$$

where the coefficient $\alpha_{i}$ is obtained by solving a convex quadratic programming problem, and $K(x,x_{i})$ is called a kernel function.

**Figure 6:**
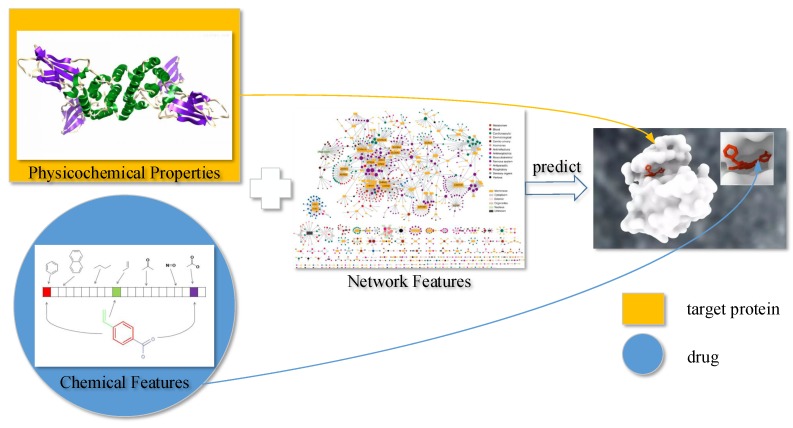
Overview of the drug–target interaction (DTI) prediction.

**Figure 7:**
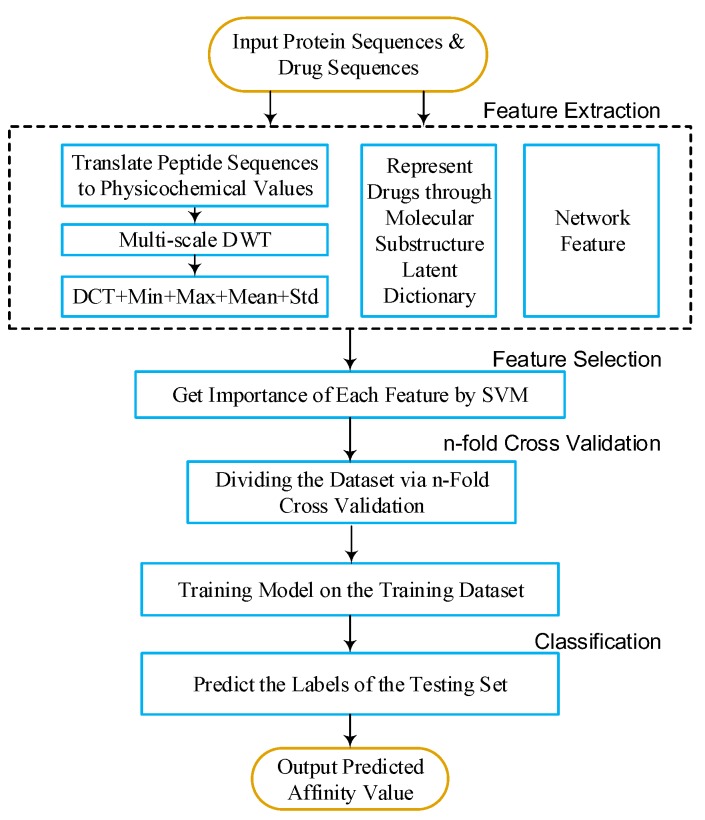
Flow chart. DWT: discrete wavelet transform; DCT: discrete cosine transform; Std: standard deviation; SVM: support vector machine.

Here, we focus on choosing a radial basis function (RBF) kernel [34], because it not only has better boundary response but can also make most high-dimensional data approximate a Gaussian-like distribution. The architecture of our proposed method is shown in Figure 6 and Figure 7.

## 5. Conclusions

In this paper, we present a DTI prediction method by using multi-scale discrete wavelet transform and network features. We employ a DWT algorithm to extract target features, and combine them with drug fingerprint and network feature. Our method can achieve satisfactory prediction performances, and our prediction can be a kind of ameliorated prediction by comparing with other existing methods after feature selection. However, the imbalanced problem of DTIs prediction is not solved very well. SVM is poor on imbalanced data. The AUPR value of DAWN is low on the Nuclear receptor dataset.

The prediction accuracy may be further enhanced with the further expansion of more refined representation of the structural and physicochemical properties or a better machine learning model (such as sparse representation and gradient boosting decision tree) for predicting drug–target interactions. In the future, we will build the classification by the strategy of bootstrap sampling and weighting sub-classifiers.

## Figures and Tables

**Table 1:** Statistics of DTI datasets [4].

	Drugs (*n*)	Targets (*m*)	Interactions	Ratio (*n*/*m*)
Enzyme	445	664	2926	0.67
IC	210	204	1476	1.03
GPCR	223	95	635	2.35
Nuclear receptors	54	26	90	2.08

IC: ion channel; GPCR: guanosine-binding protein coupled receptor.

**Table 2:** Comparison of the prediction performance between different features on balanced datasets.

Dataset	Feature	ACC	Sn	SP	AUC
Enzyme	DWT + MACCS	0.867 ± 0.002	0.861 ± 0.004	0.873 ± 0.003	0.925 ± 0.003
	DWT + MACCS (FS)	0.895 ± 0.001	0.901 ± 0.003	0.889 ± 0.003	0.949 ± 0.001
	DWT + NET + MACCS	0.932 ± 0.003	0.933 ± 0.002	0.933 ± 0.002	0.977 ± 0.002
	DWT + NET + MACCS (FS)	0.938 ± 0.002	0.938 ± 0.002	0.939 ± 0.004	0.980 ± 0.001
IC	DWT + MACCS	0.864 ± 0.003	0.868 ± 0.004	0.861 ± 0.005	0.929 ± 0.004
	DWT + MACCS (FS)	0.879 ± 0.004	0.891 ± 0.004	0.866 ± 0.007	0.935 ± 0.003
	DWT + NET + MACCS	0.940 ± 0.004	0.932 ± 0.005	0.943 ± 0.006	0.978 ± 0.003
	DWT + NET + MACCS (FS)	0.943 ± 0.002	0.938 ± 0.003	0.949 ± 0.003	0.983 ± 0.001
GPCR	DWT + MACCS	0.826 ± 0.005	0.831 ± 0.003	0.822 ± 0.007	0.872 ± 0.004
	DWT + MACCS (FS)	0.836 ± 0.006	0.846 ± 0.007	0.827 ± 0.009	0.892 ± 0.005
	DWT + NET + MACCS	0.872 ± 0.004	0.872 ± 0.005	0.872 ± 0.003	0.934 ± 0.005
	DWT + NET + MACCS (FS)	0.890 ± 0.005	0.888 ± 0.009	0.891 ± 0.011	0.950 ± 0.002
Nuclear receptor	DWT + MACCS	0.750 ± 0.011	0.619 ± 0.013	0.879 ± 0.021	0.816 ± 0.015
	DWT + MACCS (FS)	0.791 ± 0.017	0.790 ± 0.018	0.793 ± 0.036	0.850 ± 0.016
	DWT + NET + MACCS	0.805 ± 0.021	0.767 ± 0.017	0.837 ± 0.013	0.866 ± 0.011
	DWT + NET + MACCS (FS)	0.860 ± 0.009	0.855 ± 0.013	0.867 ± 0.024	0.931 ± 0.009

DWT: discrete wavelet transform; FS: feature selection; NET: network features; MACCS: drug features of molecular access system.

**Table 3:** The mean AUC values of five methods on balanced datasets.

Methods	Enzyme	IC	GPCR	Nuclear Receptor
Cao’s work [14]	0.979	**0.987**	0.951	0.924
BGL	0.904	0.851	0.899	0.843
BLM	0.976	0.973	**0.955**	0.881
NetLapRLS	0.956	0.947	0.931	0.856
RLS	0.978	0.984	0.954	0.922
DAWN (our method)	**0.980**	0.983	0.950	**0.931**

Results excerpted from [14]. The best results in each column are in bold faces. BGL: bipartite graph learning; BLM: bipartite local model; NetLapRLS: Laplacian regularized least square based on interaction network; RLS: regularized least square. DAWN: prediction of Drug–tArget interactions based on multi-scale discrete Wavelet transform and Network features.

**Table 4:** Overall AUC and AUPR values of different methods on imbalanced dataset for four species.

Evaluation	Method	Enzyme	Ion Channel	GPCR	Nuclear Receptor
AUC	NetLapRLS	0.972 ± 0.002	0.969 ± 0.003	0.915 ± 0.006	0.850 ± 0.021
	BLM-NII	0.978 ± 0.002	0.981 ± 0.002	0.950 ± 0.006	0.905 ± 0.023
	WNN-GIP	0.964 ± 0.003	0.959 ± 0.003	0.944 ± 0.005	0.901 ± 0.017
	KBMF2K	0.905 ± 0.003	0.961 ± 0.003	0.926 ± 0.006	0.877 ± 0.023
	CMF	0.969 ± 0.002	0.981 ± 0.002	0.940 ± 0.007	0.864 ± 0.026
	NRLMF	**0.987**± 0.001	0.989 ± 0.001	**0.969**± 0.004	**0.950**± 0.011
	DAWN	0.981 ± 0.004	**0.990**± 0.014	0.952 ± 0.009	0.906 ± 0.067
AUPR	NetLapRLS	0.789 ± 0.005	0.837 ± 0.009	0.616 ± 0.015	0.465 ± 0.044
	BLM-NII	0.752 ± 0.011	0.821 ± 0.012	0.524 ± 0.024	0.659 ± 0.039
	WNN-GIP	0.706 ± 0.017	0.717 ± 0.020	0.520 ± 0.021	0.589 ± 0.034
	KBMF2K	0.654 ± 0.008	0.771 ± 0.009	0.578 ± 0.018	0.534 ± 0.050
	CMF	0.877 ± 0.005	**0.923**± 0.006	0.745 ± 0.013	0.584 ± 0.042
	NRLMF	0.892 ± 0.006	0.906 ± 0.008	0.749 ± 0.015	**0.728**± 0.041
	DAWN	**0.895**± 0.011	0.921 ± 0.036	**0.786**± 0.023	0.603 ± 0.087

Results excerpted from [12]. The best results in each column are in bold faces and the second best results are underlined. BLM-NII: improved BLM with neighbor-based interaction-profile inferring; CMF: collaborative matrix factorization; KBMF2K: kernelized Bayesian matrix factorization with twin kernels; NRLMF: neighborhood regularized logistic matrix factorization; WNN-GIP: weighted nearest neighbor with Gaussian interaction profile kernels.

**Table 5:** Top five new DTIs predicted by DAWN on four data sets.

Dataset	Rank	Drug	Target	Databases
Enzyme	1	D00545	hsa1571	
	2	D03365	hsa1571	
	3	D00437	hsa1559	M
	4	D00546	hsa1571	
	5	D00184	hsa5478	D
Ion channel	1	D00542	hsa6262	
	2	D00542	hsa6263	M
	3	D00349	hsa6263	
	4	D00477	hsa6336	C
	5	D01448	hsa3782	
GPCR	1	D01051	hsa3269	
	2	D00563	hsa3269	D, M
	3	D00563	hsa1812	D
	4	D00715	hsa1129	D, K
	5	D00563	hsa1129	
Nuclear receptor	1	D01689	hsa5241	
	2	D01115	hsa5241	
	3	D00443	hsa5241	D
	4	D00443	hsa367	D
	5	D00187	hsa2099	

C: ChEMBL; D: DrugBank; K: KEGG; M: Matador.

**Table 6:** Six physicochemical properties of 20 amino acid types.

Amino Acid	H	VSC	P1	P2	SASA	NCISC
A	0.62	27.5	8.1	0.046	1.181	0.007187
C	0.29	44.6	5.5	0.128	1.461	−0.03661
D	−0.9	40	13	0.105	1.587	−0.02382
E	−0.74	62	12.3	0.151	1.862	0.006802
F	1.19	115.5	5.2	0.29	2.228	0.037552
G	0.48	0	9	0	0.881	0.179052
H	−0.4	79	10.4	0.23	2.025	−0.01069
I	1.38	93.5	5.2	0.186	1.81	0.021631
K	−1.5	100	11.3	0.219	2.258	0.017708
L	1.06	93.5	4.9	0.186	1.931	0.051672
M	0.64	94.1	5.7	0.221	2.034	0.002683
N	−0.78	58.7	11.6	0.134	1.655	0.005392
P	0.12	41.9	8	0.131	1.468	0.239531
Q	−0.85	80.7	10.5	0.18	1.932	0.049211
R	−2.53	105	10.5	0.291	2.56	0.043587
S	−0.18	29.3	9.2	0.062	1.298	0.004627
T	−0.05	51.3	8.6	0.108	1.525	0.003352
V	1.08	71.5	5.9	0.14	1.645	0.057004
W	0.81	145.5	5.4	0.409	2.663	0.037977
Y	0.26	117.3	6.2	0.298	2.368	0.023599

H: hydrophobicity; VSC: volumes of side chains of amino acids; P1: polarity; P2: polarizability; SASA: solvent-accessible surface area; NCISC: net charge index of side chains.
